# A Study on the Nature of SARS-CoV-2 Using the Shell Disorder Models: Reproducibility, Evolution, Spread, and Attenuation

**DOI:** 10.3390/biom12101353

**Published:** 2022-09-23

**Authors:** Gerard Kian-Meng Goh, A. Keith Dunker, James A. Foster, Vladimir N. Uversky

**Affiliations:** 1 Goh’s BioComputing, Singapore 548957, Singapore; 2 Center for Computational Biology, Indiana and Bioinformatics, Indiana University School of Medicine, Indianapolis, IN 46202, USA; 3 Department of Biological Sciences, University of Idaho, Moscow, ID 83844, USA; 4 Institute for Bioinformatics and Evolutionary Studies, University of Idaho, Moscow, ID 83844, USA; 5 Department of Molecular Medicine, Morsani College of Medicine, University of South Florida, Tampa, FL 33612, USA

**Keywords:** coronavirus, COVID, intrinsic, disorder, nucleoprotein, Omicron, pangolin, shell, virulence, long COVID, attenuation, variant

## Abstract

The basic tenets of the shell disorder model (SDM) as applied to COVID-19 are that the harder outer shell of the virus shell (lower PID—percentage of intrinsic disorder—of the membrane protein M, PID_M_) and higher flexibility of the inner shell (higher PID of the nucleocapsid protein N, PID_N_) are correlated with the contagiousness and virulence, respectively. M protects the virion from the anti-microbial enzymes in the saliva and mucus. N disorder is associated with the rapid replication of the virus. SDM predictions are supported by two experimental observations. The first observation demonstrated lesser and greater presence of the Omicron particles in the lungs and bronchial tissues, respectively, as there is a greater level of mucus in the bronchi. The other observation revealed that there are lower viral loads in 2017-pangolin-CoV, which is predicted to have similarly low PID_N_ as Omicron. The abnormally hard M, which is very rarely seen in coronaviruses, arose from the fecal–oral behaviors of pangolins via exposure to buried feces. Pangolins provide an environment for coronavirus (CoV) attenuation, which is seen in Omicron. Phylogenetic study using M shows that COVID-19-related bat-CoVs from Laos and Omicron are clustered in close proximity to pangolin-CoVs, which suggests the recurrence of interspecies transmissions. Hard M may have implications for long COVID-19, with immune systems having difficulty degrading viral proteins/particles.

## 1. Introduction

### 1.1. The Enigmas of COVID-19-Related Viruses and SARS-CoV-2

Officially, the first cases of the coronavirus disease 2019 (COVID-19) were noticed in Wuhan, China in December 2019 [1,2,3,4]. The causative agent, Severe Acute Respiratory Syndrome Coronavirus-2, SARS-CoV-2, as it was later named, was quickly identified as the culprit and its genetic sequence was published. SARS-CoV-2 is about 80% genetically similar to the 2003 SARS-CoV (SARS-CoV-1). A retrospective search determined that a genetic sequence of a bat coronavirus sample, RaTG13, taken from a cave in Yunnan in 2013, has 96.2% similarity with that of SARS-CoV-2 [5,6]. Later, a series of coronavirus samples were taken from bats in Laos, and one sample, BANAL52, has a sequence homology of 96.8% in comparison to the SARS-CoV-2 sequence [7].

Two sets of CoV samples were obtained from pangolins that were confiscated from smugglers in Guangdong and Guangxi provinces. The samples from Guangxi were confiscated in 2017–2018, whereas the ones from Guangdong were in 2019 [8,9,10,11,12]. The samples have about 90% sequence homology with SARS-CoV-2. Recently, it was discovered that pangolins confiscated in Vietnam around 2017–2018 were also infected with a COVID-19-related virus [13]. Phylogenetic analysis revealed that the CoV is most closely related to the pangolin samples from Guangxi. The mystery involving the proximal origin of SARS-CoV-2 lingers. Even more questions arose with the arrival of the Omicron variant of SARS-CoV-2.

The “original” Wuhan-Ju-1 strain struck around December 2019, but many variants, including Alpha, Beta, Gamma, and Omicron, prevailed at various times thereafter [14]. Arguably, the most intriguing is the Omicron variant. It was first observed in South Africa among patients that showed mainly mild symptoms. In contrast to Delta and all other variants known then [4,15,16], significantly fewer hospitalized patients needed oxygen ventilators, and much lower numbers of patients were placed in the intensive care units [15,16]. Even though the evidence shows that this variant is a milder virus, there are debates involving the question whether the Omicron’s milder clinical outcome is something intrinsic to the virus, or, rather, just a manifestation of a more vaccinated population [17].

Furthermore, Omicron is the most mutated variant found thus far, with over 50 mutations [18]. Many scientists are puzzled by such a high level of mutations, given that CoVs tend to mutate less easily than other RNA viruses as their RNA-polymerase prevents too many mutations, unlike many other viruses [19,20]. This enigma assumes that Omicron is a descendant of Wuhan-Hu-1. Some of our previous papers showed that this assumption may not be correct [21,22]. Additionally, many of the mutations seen in Omicrons are different from all other variants. The question is then: Where was Omicron hiding all along? Some have hypothesized that it had been hiding in the bodies of HIV-infected individuals that are immune-compromised [23]. Others believe that it has been hiding in animals, such as mice. We have previously reported that our shell disorder models (SDMs) provide evidence that a burrowing animal, such as pangolins, could provide the optimal environment for the virus to incubate and evolve into a milder but more transmissible variant [21,22]. We will see that SDMs have specific answers or hints to many of the mysteries set forth.

### 1.2. COVID-19 and the Shell Disorder Models (SDMs)

A set of models, SDMs, were developed to understand the viruses in general, with the parent shell model being first published in 2008, as seen in Table 1 [24,25,26]. SDMs involve the use of artificial intelligence to measure the levels of intrinsic disorder in the viral shell proteins.

The parent SDM, the viral shape-shifting model, detected unusually large levels of disorder in the outer matrix shell of many isolates of HIV-1, which are likely to contribute to the repeated failures in the search for an effective HIV vaccine [24,25,26,27,28]. A daughter SDM was developed and first published in 2012 [29]. This model, the CoV transmission SDM, correlated the levels of the fecal–oral and respiratory transmission potentials of CoVs to the levels of intrinsic disorder in M and N proteins found in their two shells. A second SDM was first published in 2015 [26,30], using correlations that were found between the inner shell percentage of intrinsic disorder (PID) and the virulence of a variety of viruses, including dengue virus (DENV), Ebola virus (EBOV) and Nipah virus (NiV) [26,30,31,32,33].

When COVID-19 struck, the SDMs were put to work and were able to reproduce the characteristics of the virus and disease that were later observed clinically and experimentally [22,34,35,36,37]. SDM analysis of the protein sequence of the Wuhan-Hu-1 strain using the CoV-transmission model suggested that this virus falls into the category of CoVs with intermediate fecal–oral and respiratory potentials, but the SDM analysis detected something abnormal in this virus that is not seen in virtually all CoVs we have studied in the past. SARS-CoV-2 has one of the hardest outer shells (lowest PID_M_) within its CoV family [36,37]. Subsequent experiments have re-affirmed that SARS-CoV-2 lasts longer than many CoVs away from ultraviolet light [38]. Upon further database search, it was found that such hard M is associated with burrowing animals such as rabbits and with buried feces [22,35,37]. The discovery of pangolin-CoVs reinforces this observation, as pangolins are burrowing animals and pangolin-CoVs have hard M just like all COVID-19 related viruses.

The hard outer shell is important; it provides a clue to the reason why SARS-CoV-2 is highly transmissible among humans because it is more capable of resisting the anti-microbial enzymes found in saliva and mucus [36,39,40,41]. If we assume that this hypothesis is correct, SDMs make certain bold predictions, especially with regard to viral spread, that could easily be verified. Similarly, the virulence-inner shell disorder model makes specific predictions if we assume that it is applicable to COVID-19. While we were not sure if it could be applied to COVID-19, as we had insufficient data, the higher PID_N_ (PID_N_ = 50%) and case–fatality ratio (CFR~10%) of SARS-CoV-1 in comparison to those of SARS-CoV-2 (PID_N_ = 48%; CFR = 2%) made us suspect that this model is applicable in these cases as well. As more data arose from laboratories and clinics throughout the world, it became clear that the predictions made by SDMs are accurate.

One prediction by the SDMs was that a pangolin-CoV sample collected in Guangxi in 2017 is attenuated, and that if it had entered the human population, it could have silently spread, as it could easily be dismissed as a mild cold by the medical community [35]. This was based on the low PID (PID_N_~45%) obtained from an analysis of the N protein sequence. Furthermore, we predicted that the pangolin-CoV 2017 spread may be somewhat slower because its outer shell is slightly less disordered than that of SARS-CoV-2, while its PID_N_ is much lower than that of SARS-CoV-2.

Omicron first struck southern Africa and later spread rapidly throughout the world. Since its early days, physicians and scientists have noticed that the virus generally manifests milder symptoms but spreads faster than the other variants. Upon inspection of the PID_N_ and PID_M_ of Omicron, it became clear that our predictions were consistent with what we were seeing with the Omicron variant [22]. The Omicron PID_N_ resembles that of the pangolin-CoV, which could account for its attenuation, while its PID_M_ is distinctly lower than those of other SARS-CoV-2 variants and that of pangolin-CoV-2017, which could also account for its quicker spread. Apparently, the SDMs’ results are providing specific answers to many of Omicron’s enigmas.

We are not the only group that has produced evidence of the intrinsic attenuation of Omicron. For example, researchers from Hong Kong University (HKU) Medical School were able to extract bronchia l and lung tissues separately to infect them with the various SARS-CoV-2 variants [42,43]. Data pertaining to the viral titers indicate a greater presence of virus copies in the bronchial tissues than lung tissues in the case of Omicron in comparison to other variants [42,43]. In a previous study, we showed that there were correlations between the mentioned results and PID_N_ and PID_M_ values. However, at the time of the writing of the previous paper, [43] was yet to be published and only partial data were released [42]. As a result, the correlations presented in our previous study were not statistically significant [37]. In this paper, correlations with statistical significance are presented using more complete data available from [43]. Furthermore, data showing the viral titer of pangolin-CoV-2017 in comparison to that of SARS-CoV-2 were not available then [44,45]. Additionally, shell disorder analysis was yet to be performed on the COVID-19-related sequence obtained from bats in Laos [7]. Previously, phylogenetic trees using M have been shown to be different from others. We argue that phylogenetic study offers the most accurate data because M is likely to be more conserved than any other COVID-19 proteins as a result of the mentioned crucial role it plays among burrowing animals and buried feces. Most phylogenetic algorithms are unable to effectively handle recombinations that could mislead many important phylogenetic studies [46]. The Laotian samples were studied using phylogenetic analysis of the M protein in this paper for the first time.

## 2. Materials and Methods

SDMs are based on the concept of protein intrinsic disorder, which is defined as a lack of unique structure in part of the whole functional protein [47,48,49,50,51,52,53,54,55,56,57,58,59,60,61,62]. Multiple computational tools have been developed to predict disorder. These tools are based on past empirical knowledge of proteins that were experimentally characterized as disordered. The knowledge arose from experiments using X-ray crystallography and several other biophysical approaches. The first tool for sequence-based prediction of the predisposition of a query protein for intrinsic disorder, and one that continues to be broadly used in research, was PONDR^®^-VLXT [63,64]. This tool was also utilized in all studies conducted by our group, where it was applied o analyze a large variety of viruses, such as DENV, EBOV, NiV, Influenza, yellow fever virus, and CoVs [21,22,24,25,28,29,30,31,32,33,34,35,36,37,65,66,67]. The choice of this tool was determined by its high sensitivity to local sequence peculiarities resulting in the high sensitivity for detection of potential disorder-based interaction sites undergoing disorder-to-order transition on binding to specific partners, such as other proteins, glycoproteins, RNA and DNA [68,69,70,71]. PONDR^®^-VLXT is a neural network that predicts order and disorder predisposition at each residue based on the respective protein sequences inputted [63,72,73]. The sequences of the viral proteins were searched, carefully examined and downloaded from either UniProt [74] or NCBI-Protein/GenBank [75]. The outputs and the sequence data were placed in a MYSQL database [76] using JAVA programming language m [77]. An important measure of the level of disorder in query proteins is its percentage of disorder (PID). PID is defined as the number of residues predicted to be disordered divided by the total number of residues in a query protein times 100.

The phylogenetic trees were derived from software available at the EMBI-EBI website [78] and the TREX website [79], with the algorithms used being CLUSTAL OMEGA and CLUSTALW, respectively. Further annotations of the trees, such as PIDs, were added using GIMP [80]. The schematic diagram was drawn using OpenOffice [81] and GIMP [80]. The sequence similarities were obtained using NCBI-BlastP [82]. The experimental data from HKU can be directly downloaded from the supplementary section of the paper, whereas the data points for the pangolin-CoV experiment have to be interpolated from the figures using a ruler. Multivariate analyses were performed using R [83].

## 3. Results

### 3.1. The CoV-Transmission-Shell Disorder Model

In 2012, before the MERS-CoV outbreak in the Middle East, the first CoV transmission SDM paper was published [29,84]. It was based on a statistical study of disorder at M and N proteins as applied to knowledge of the behaviors of animal coronaviruses, with particular attention given to porcine CoVs. Prior to the 2003 SAR-CoV outbreak, coronaviruses were considered unimportant by the medical community, as they were usually associated with mild cold symptoms then. The case is different in veterinary science, as it is not uncommon for animal coronaviruses, such as TGEV (transmissible gastroenteritis virus), to devastate farming communities.

Using statistical analysis, the original model was able to group coronaviruses into three identifiable groups (ANOVA, *p* < 0.05), similar to those shown in Table 2, with the exception that category D, which is made up of CoVs with hard outer shell, was not included in the original model. As we will see later, this omission was inevitable, given the lack of samples in Category D before the COVID-19 outbreak. Category A consists of CoVs with higher PID_N_ values that are considered to have higher respiratory transmission but lower fecal–oral potentials. CoVs in category B are those with intermediate fecal–oral and respiratory transmission potentials, along with medium levels of PID_N_ values, whereas category C includes CoVs with higher fecal–oral but lower respiratory transmission potentials.

**Figure 1:**
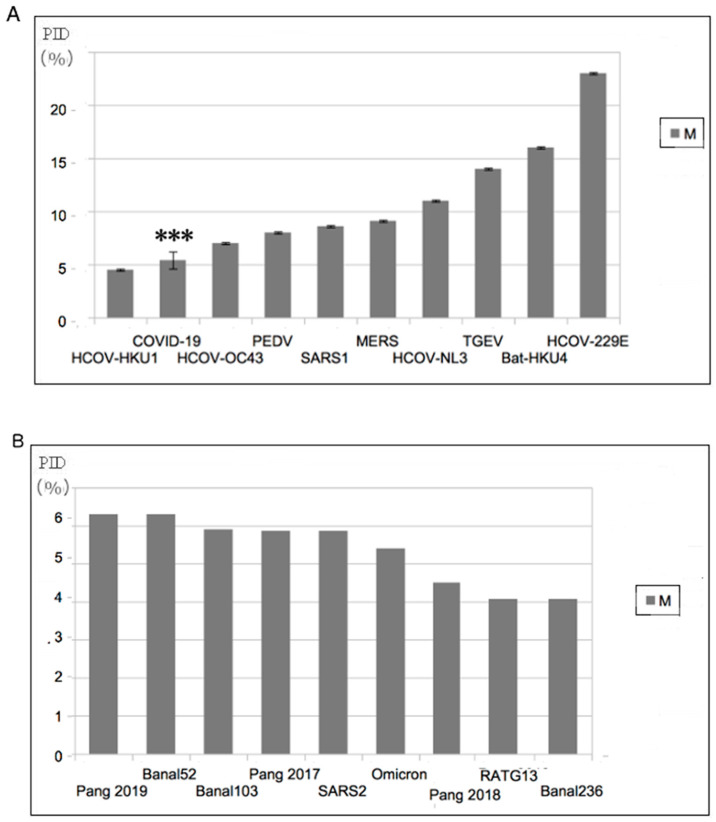
Abnormally Hard Outer Shell (Low PID_M_) of COVID-19-Related Viruses (**A**) All known COVID-19 viruses have among the hardest outer shells within the CoV family. “***” refers to SARS-CoV-2. (**B**) Comparison of PID_M_ values among COVID-19 related viruses. A relatively harder M can be found in Omicron. The label “SARS2” in (**B**) refers to all non-Omicron SARS-CoV-2 variants currently known. (Taken in part from Biomolecules. 2022;12(5). © 2022 Goh et al, Published by MDPI AG, Open access under CC BY-NC-ND license. This is an Open Access article which permits unrestricted non-commercial use, provided the original work is properly cited. [21]).

While the original model correlated the modes of transmission mainly with PID_N_, the relationship with PID_M_ was more enigmatic right from the beginning. A regression analysis using N as the sole independent variable yielded a coefficient of determination (r^2^) of 0.77 but a slightly yet noticeably stronger correlation was seen when PID_M_ was added as another independent variable (r^2^ = 0.83). While statistics tells us that PID_M_ is an essential co-independent variable to PID_N_, the exact role of M in the mode of transmission remained elusive until data started pouring in from COVID-19. We will note that in the list of CoVs, with the exception of COVID-related viruses, there are very few, if any, viruses that have low PID_M_ values. Figure 1 allows us to emphasize this point. Figure 1A shows that with the exception of CoV-HKU1, no other CoV has a lower PID_M_. Figure 1B shows the consistency of the low PID_M_ across all COVID-19 viruses.

### 3.2. Abnormally Hard Outer Shell (Low PID_M_) Rarely Seen Outside of COVID-19-Related CoVs

The abnormally hard outer shell (low PID_M_) was the first thing that struck us when we first inspected the COVID-19 proteins. We immediately suspected that this hard outer shell has something to do with the high contagiousness of SARS-CoV-2, as we have previous data from other viruses indicating that hard outer shells are often related to resistance to the anti-microbial enzymes found in saliva and mucus. Based on incoming experimental, clinical and computational COVID-19 data, we now know that the harder outer shell of SARS-CoV-2 is able to shed itself in larger quantities and higher concentrations orally and nasally because of its unique hardness, which allows for greater resistance against anti-microbial enzymes. The fact that Omicron has an even harder outer shell but is even more infectious than all other variants thus far, including the Wuhan strain, reproduces the prediction made by SDMs. This is just one of the many predictions made by the SDMs that have been reproduced experimentally.

### 3.3. Abnormally Hard Outer Shell (Low PID_M_) Evolutionarily Arose from a Burrowing Animal Such as Pangolins via Buried Feces

Where did this characteristic of hard outer shell come from? The answer can be found through a careful inspection of Table 2. While there are very few CoVs with such low PID_M_ values, the few that we could find are all associated with burrowing animals, such as rabbits and pangolins. HCoV-HKU1 has a hard outer shell and is phylogenetically close to that of mice [85,86]. Mice have dual evolution that involves living in burrows and human homes, respectively [87]. Given these, it is likely that the hard outer shell is associated with fecal–oral transmission via buried feces. It is also intriguing to notice that while pangolin-CoVs are related to SARS-CoV-2, rabbit-CoV and HCoV-HKU1 are not. A glance at the data in Table 3 tells us that pangolin-CoVs offer a relationship to SARS-CoV-2 not seen with any other COVID-19 viruses. Pangolin-CoVs provide a range of PID_N_ values that are most similar to those of the SARS-CoV-2 variants, especially Omicron. In contrast, COVID-19-related bat-CoVs remain constant at around 48% for PID_N_, with none matching the PID_N_ of Omicron.

### 3.4. Omicron Has a Lower PID_N_ Similar to That of Pangolin-CoV-2017 but Has a Lower PID_M_: Attenuation and Faster Spread

Before the outbreak of Omicron, we analyzed the PID_N_ and PID_M_ of the various pangolin-CoVs and compared them to those of SARS-CoV-2. The similarities and differences are so striking that we were able to pinpoint that the pangolin-CoV from 2017 was likely to be attenuated, and if it had entered the human population, it would have been clinically manifested by symptoms of a mild cold that could easily have gone undetected. We also predicted that the virus is likely to spread more slowly among humans, since its PID_M_ is almost identical to that of SARS-CoV-2, whereas its PID_N_ is much lower. When Omicron came, it was discovered that this variant is relatively mild, and we found that the N disorder of Omicron is almost identical to that of pangolin-CoV-2017. Just as interestingly, Omicron is more infectious than any other variants known thus far, and its PID_M_ is lower than that of pangolin-CoV-2017 and any known variants. Summarily, Omicron has reproduced two important but different predictions of the SDMs.

**Figure 2:**
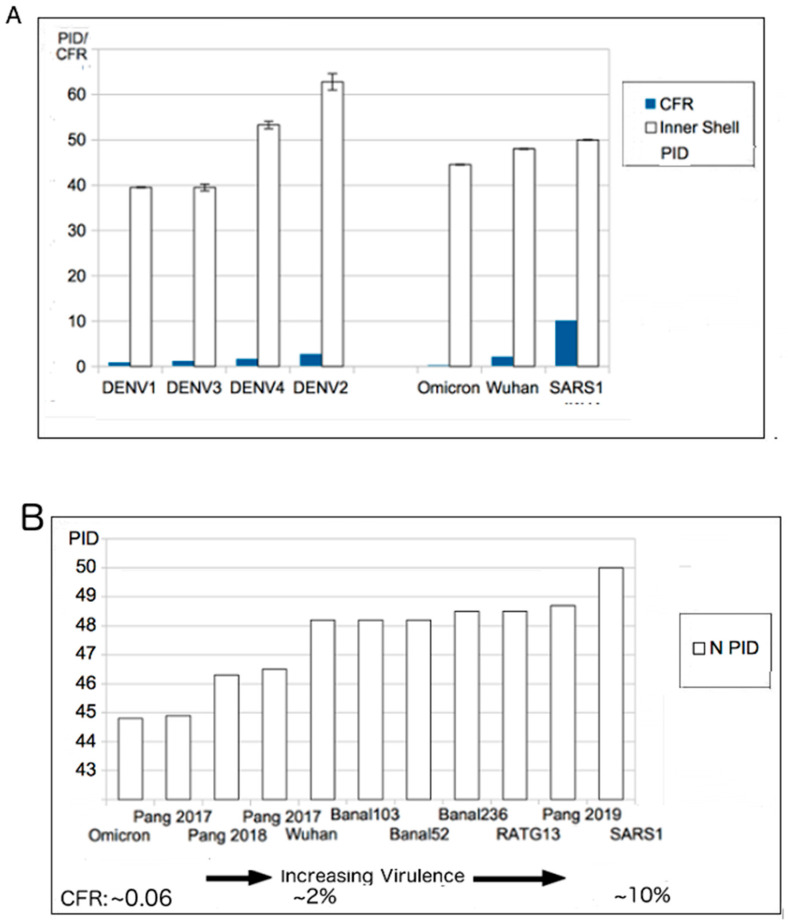
Virulence-Inner Shell Disorder Model as Applied to DENV and COVID-19. (**A**) Correlation of DENV virulence with inner shell (NP) disorder (r = 0.95) [26]. (**B**). Correlation between SARS-related viruses and PID_N_. The correlation is based on estimated case fatality ratios (CFRs) of SARS-CoV-1/2 and Omicron. The two values for Pang 2017 (pangolin-CoV 2017) are based on the two separate isolates collected in 2017. Further details on the accession codes can be found in Table 3. (**B**): Taken in part from Biomolecules. 2022;12(5). © 2022 Goh et al., Published by MDPI AG, Open access under CC BY-NC-ND license. This is an Open Access article which permits unrestricted non-commercial use, provided the original work is properly cited. [21]).

The prediction of attenuation arose from the virulence-inner shell disorder model, which basically involves a strong correlation between virulence and inner shell disorder in many viruses, such as NiV, DENV and EBOV. Examples can be seen in Figure 2. As in Figure 2A, the DENV C (inner shell) PID has a high correlation (r^2^ = 0.92) with the virulence, whereas SARS-CoV-2 has also shows a high correlation (r^2^ = 0.8) between its PID_N_ and case fatality ratio (CFR). Prediction of potential human virulence can be extended to COVID-19-related viruses using PID_N_, as seen in Figure 2B.

### 3.5. The Role of N in CoV Transmission SDM and Virulence-Inner Shell Disorder Model

As we have seen, N protein plays important roles in both CoV-transmission SDM and the Virulence-Inner Shell Disorder model. Inner shell proteins have been known to assist the replication of many viruses. Their functions including the assembly of viral proteins and RNA/DNA for packaging and release of viral particles. Such inner shell proteins often play a role in binding to the viral RNA/DNA during the replication of the genetic material. Disorder in the inner shell proteins provides for more efficient binding. CoV N is known to play roles in the packaging of the viral genome during replication, and we have previously found that there are lower levels of disorder in the RNA binding regions of both 2017 pangolin-CoV and Omicron N proteins, in comparison with other SARS-CoV-2 variants [37,88].

The reason for the high correlation between mode of transmission and N disorder has to do with the necessity of the shedding, nasally and orally, of viral particles at sufficiently high levels for respiratory transmission to be even possible. As a result, the greater the N disorder is, the greater the viral load in the saliva and mucus, assuming that the PID_M_ values are approximately constant across viruses and not radically different, as in the case of SARS-CoV-2.

In the case of the virulence-inner shell disorder model, the same logic holds except that it is applied to vital organs of the host. The more disordered the viral inner shell is, the more efficiently the virus is reproduced in vital organs, which have a greater probability of failure if the viral load becomes too immense at their respective locations. The presence of such a mechanism of virulence has to do with a “Trojan Horse” immune evasion strategy, in which a virus will attempt to replicate rapidly before the host immune system is able to recognize its presence and then attempt to eliminate it. In doing so, this strategy often backfires on the virus by killing the host.

### 3.6. Phylogenetic Trees Using M Offer the Best Snapshot: M Is Highly Conserved

**Figure 3:**
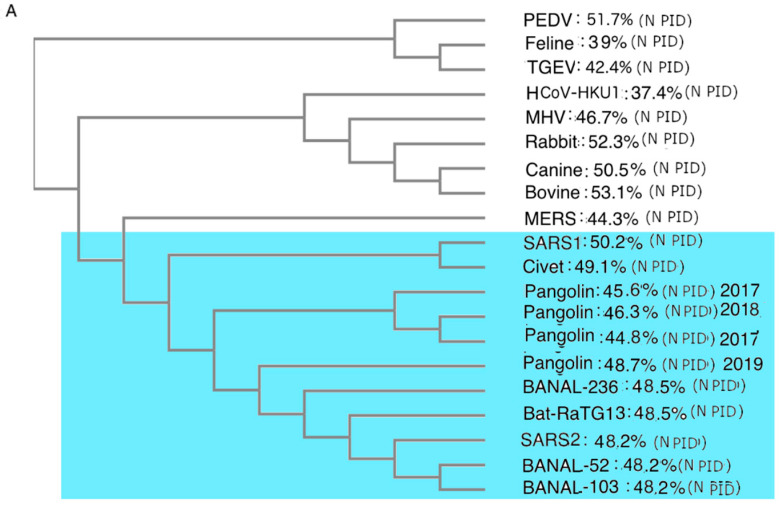

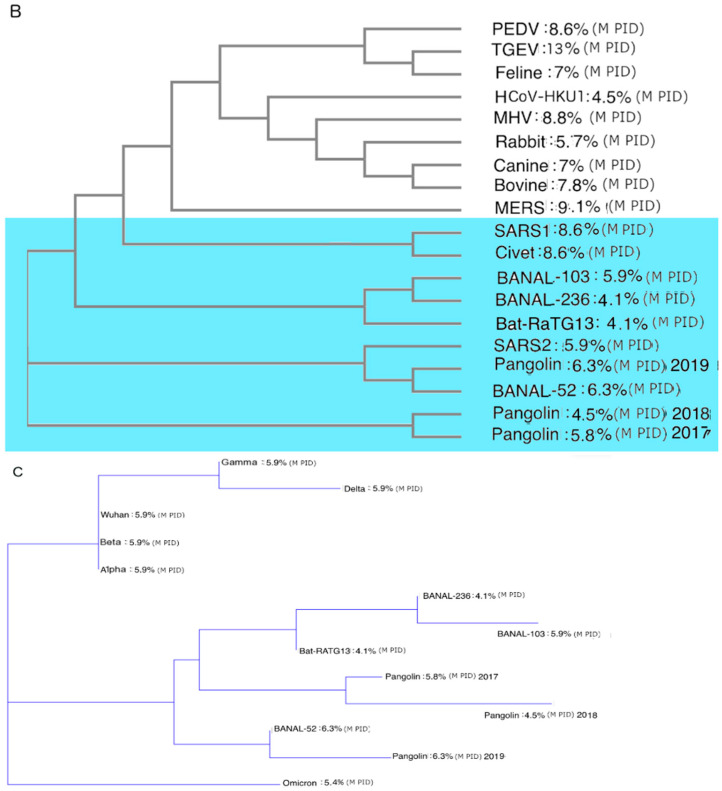
CoV Phylogenetic Trees with PID_N_ and PID_M_ Values. (**A**) Phylogenetic trees of CoVs using N proteins. Analysis was performed using CLUSTAL OMEGA [78]. (**B**) Phylogenetic trees of CoVs using M. Analysis was performed using CLUSTAL OMEGA [78]. (**C**) Phylogenetic trees of COVs related to SARS-CoV-2 using M protein. Analysis was performed using CLUSTALW [79]. Blue region denotes viruses related to SARS-CoV-2.

While phylogenetic studies of COVID-19-related viruses have been conducted previously, our group has offered a uniquely different approach. While most research has focused on phylogenetic studies of the COVID-19-related viruses using the S protein or genome-wide sequence, we use the M protein. This is because we believe that the phylogenetic tree built using M offers the most accurate snapshot of the evolution of the COVID-19-related viruses, as M is highly conserved, as seen in Table 3. The rigid structure of the M protein forces it to be conserved and, as a result, there is less chance of recombination occurring, unlike other viral proteins. Most phylogenetic algorithms handle recombination poorly, and this issue may have misled many current COVID-19 research efforts. Figure 3 contrasts phylogenetic trees using M to one that uses N. Most COVID-19 phylogenetic trees resemble Figure 3A, in contrast to Figure 3B,C. One major difference between Figure 3A–C is the positions of the pangolin-CoVs and SARS-CoV-2. Figure 3B,C shows that pangolin-CoVs have a greater relationship to SARS-CoV-2 than to most COVID-19 viruses. Previously, we published similar phylogenetic trees without the Laotian bat-CoVs. Figure 3B,C, however, shows also a close relationship between the Laotian bat-CoVs and pangolin-CoVs. Intriguingly, Figure 3C shows that Omicron may be closer to pangolin-CoVs and bat-CoVs than some of the other SARS-CoV-2 variants. This provides further support to the hypothesis that Omicron had been hiding in a burrowing animal all these years before surfacing among humans.

### 3.7. Closer Relationship between SARS-CoV-2 and Pangolin-CoV

In previous studies, we have shown that pangolin-CoVs are more closely related to SARS-CoV-2 based on the phylogenetic trees using M and their PID_N_ and PID_M_ values. These studies did not consider the Laotian COVID-19-related bat-CoVs. With the Laotian bat-CoV data, we are able to see that the Laotian bat-CoVs cluster closely with the pangolin-CoVs. This is consistent with what is known about the behaviors of both bats and pangolins, which often live together in close space and are, therefore, likely to infect each other with viruses. There are, however, noticeable differences in the molecular trends and characteristics of the viral proteins found in the two animals. More specifically, the PID_N_ values of the pangolin-CoVs are more diverse than those of COVID-19-related bat-CoVs. From Table 2 and Table 3 and Figure 3, we are able to see that the pangolin-CoV PID_N_ values have a range of 44–48%, whereas the PID_N_ of COVID-19 bat CoV remains around 48% even with the larger size of the Laotian sample. A hint of the reason for this disparity can be seen in Table 2 and Table 3, which tell us that bat-CoV PID_N_ values tend to fall in a narrow range even if they are non-COVID-19-related.

This is likely why the behavior of bats plays a role in the spread of CoVs, where a certain level of respiratory potential is necessary, perhaps, because bats are flying animals. A pangolin is a burrowing animal that flicks its sticky tongue to eat ants. This behavior inevitably leads to swallowing of feces, particularly buried feces, as pangolins have strong arms that can dig for subterranean termites [35]. We will see that this behavior has implications for the lifecycle of SARS-CoV-2 and its human spread.

### 3.8. Phylogenetic Trees Suggest Decades of Interspecies Transmission

The intermingled ancestry of pangolin, human and bat COVID-19-related viruses (Figure 3B,C) suggests that the virus has been moving to and from the species for years, if not decades. Evidence that pangolins and bats are often in close proximity has been seen [89]. The repetitive interspecies transmission may provide insight into the mechanism by which SARS-CoV-2 acquires its unusual multi-species adaptation [90]. It also reiterates the suggestion made in our previous publications that an attenuated COVID-19-related virus may have entered the human population in 2017 if not earlier. An entry of an attenuated virus could easily have been mistaken as a mild cold by the medical community, especially if the spread was slow.

### 3.9. SARS-CoV-2’s Evolution within Animals Affects Its Virulence and Human Spread

The evolutionary pressure towards fecal–oral transmission in pangolins forces the virus not just to maintain its harder outer shell, but it also adds to pressure for the virus to acquire a harder inner shell, as all shells play roles in protecting the virion from damage even if the outer shell plays a larger role. Both aspects of the evolutionary pressure have been observed in other viruses in the past. For example, insect-borne viruses are often held at the mouth of the insect, where they are exposed to the anti-microbial enzymes found in the saliva, and virtually all such viruses have hard outer shells, as in the case of flaviviruses. Some of them have noticeably hard inner and outer shells, such as EIAV and rabies virus (rabies virus is not insect-borne but dwells near the salivary glands of animals) [24,25,26,29,30].

**Figure 4:**
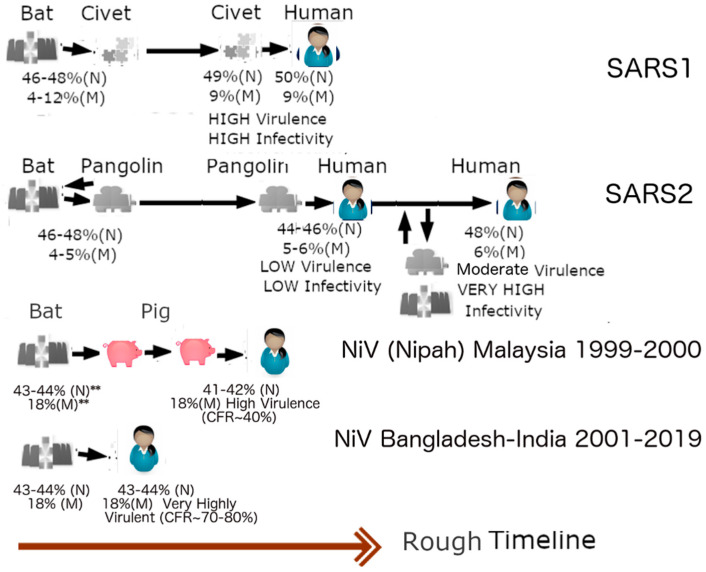
Zoonotic Relationships and Virulence. The introduction of SARS1 (SARS-CoV-1) into humans may be linked to an intermediary involving civet cats. Because of the higher virulence of SARS1, in comparison to SARS2 (SARS-CoV-2), and the high genetic similarity of SARS-CoV1 and Civet-CoV, it can be suspected that SAR1 entered civets for a short while before entering humans. The lower virulence of SARS-CoV-2 leads us to believe that the virus may have been incubating in an intermediary involving a burrowing animal such as pangolin for a relatively longer period of time. The case of Nipah virus illustrates this point. The virus involved in the 1999–2000 Malaysian outbreak is less virulent than the viruses involved in outbreaks in Bangladesh and India because the former involved an intermediary, i.e., farm pigs. The reason that SARS-CoV-1 (SARS1) was more virulent is likely that it was in an intermediary host (palm civet cat) for a relatively short period. SARS-CoV-2, (SARS2), unlike SARS1, could have been incubating among pangolins for a long time before its arrival among humans as a relatively more attenuated virus, in comparison to SARS1e. Omicron is likely to have arisen from a backward transmission to a burrowing animal such as pangolin. It is also possible that a non-Omicron variant re-entered the pangolin population already infected with Omicron and recombinations occurred. (Taken in part from Biomolecules. 2022;12(5). © 2022 Goh et al, Published by MDPI AG, Open access under CC BY-NC-ND license. This is an Open Access article which permits unrestricted non-commercial use, provided the original work is properly cited. [21]).

As mentioned above, a harder outer shell is more resistant to the anti-microbial enzymes found in the saliva and mucus, whereas a harder inner shell leads to the production of fewer copies of viral particles, particularly in vital organs. Figure 4 provides a summary of the evolutionary significance of the described properties. SARS1 (2003 SARS-CoV-1) has higher PID_N_ and PID_M_ values than SARS-CoV-2 (SARS2). This is consistent with the fact that SARS1 is more virulent than SARS2. A question, then, is: What is the evolutionary mechanism that can account for this difference? According to the SDMs, the SARS1 virus is likely to have stayed in an intermediary animal, such as the palm civet cat for not too long after a transmission from bats. Since bat-CoVs tend to have high PID_N_ values, it is likely that the PID_N_ during the interspecies transmissions remained mostly unchanged. SARS2, on the other hand, is much less virulent than SARS1. SARS2 could have been attenuated, as the virus had been incubating in pangolins, as pangolins provide a more optimal environment for attenuation by their fecal–oral behaviors. It is also likely that pangolins have been a reservoir for COVID-19 viruses for a relatively long period of time. Some could, however, argue against such a scenario by pointing to the similarity of bat-CoV-2 PID_N_ values to non-Omicron SARS2 variants. Such arguments are, however, oblivious of the probability of transmission going to and fro between pangolins and bats on a regular basis. Figure 4 also points to the example of NiV, which exhibits a much higher virulence (CFR: 70–80%), in contrast to a NiV variant that involved a porcine intermediary (CFR~40%), as in the case of the Malaysian outbreak in 1999–2000 [32,91].

### 3.10. The Evolution of SARS-CoV-2 and Omicron Have Important Implications for the Lifecycle and Clinical Manifestations of COVID-19

**Figure 5:**
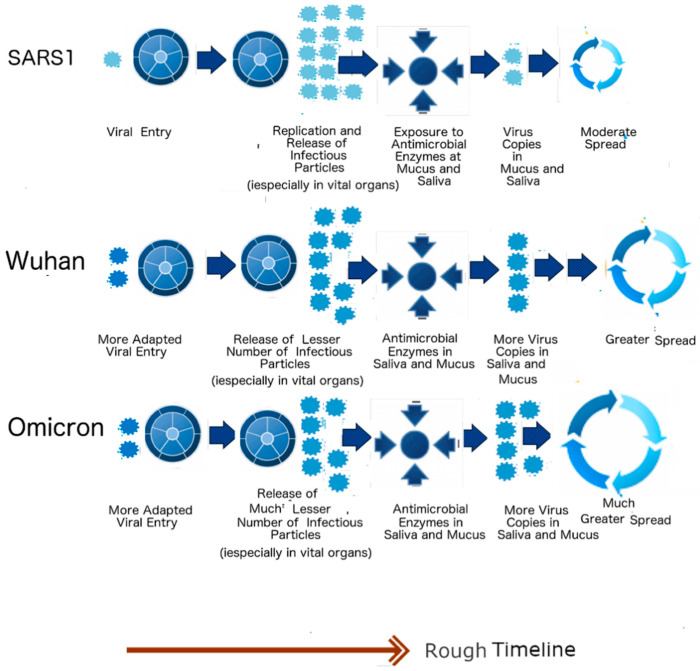
Lifecycles of SARS-CoV-1, Wuhan-Hu-1 Strain and the Omicron Variant. The schematic diagram summarizes implications arising from the SDMs. The higher PID_N_ allows greater production of viral particles in the lungs, but the softer outer shell (lower PID_M_) causes less viral shedding in the oral–nasal region when compared to SARS-CoV-2. (Taken in part from Biomolecules. 2022;12(5). © 2022 Goh et al, Published by MDPI AG, Open access under CC BY-NC-ND license. This is an Open Access article which permits unrestricted non-commercial use, provided the original work is properly cited. [21]).

Right from the beginning of COVID-19, careful clinical studies have been conducted. It was found that COVID-19 patients shed larger quantities of viral particles than those infected with SARS-CoV-1 [92]. The lifecycle of SARS-CoV-2 is also longer, with some patients shedding particles even after 6 months [92]. SDMs have a specific and consistently reproducible explanation for both the increase in virulence, longer lifecycle and greater spread of SARS-CoV-2. The higher shedding is due to the greater hardness of the SARS-CoV-2 outer shell (low PID_M_), which causes it to be more resistant to the anti-microbial enzymes found orally and nasally. It is also for this reason that some patients are able to keep shedding viral particles for a longer period. As already mentioned, the lower virulence of SARS-CoV-2 is a result of the lower PID_N_ (48% vs. 50%). This has been proven to be true experimentally using Vero E6 cells that produce more SARS-CoV-1 particles than SARS-CoV-2 [93]. Figure 5 summarizes the clinical effects of the mentioned properties. In the case of SARS1 (SARS-CoV-1), more particles are produced in the body, especially in the vital organs such as lungs, but by the time the virus reaches the nose and mouth, many of the particles have been eliminated by the microbial enzymes. In contrast, SARS-CoV-2, as represented by Wuhan-Hu-1 (Wuhan strain), produces fewer copies in the vital organs, but the body manages to shed more viral particles orally and nasally.

When Omicron arrived, it became yet another opportunity to validate the SDMs. Clinical studies have shown that Omicron is milder and more infectious than any other SARS-CoV-2 variant. Yet again, the predictions of the SDMs were proven to be highly reproducible, with both PID_M_ and PID_N_ of Omicron shown to be lower than those in all other variants. According to the SDMs, Omicron will produce even fewer viral particles in vital organs but shed even more virus copies, as seen in Figure 5. It should also be noted that SARS-CoV-2 has furin cleavage sites at the S protein, unlike SARS1. This (as illustrated by the two viral particles at the viral entry in Figure 5) allows for more efficient viral entry, but it cannot account for the other characteristics mentioned.

### 3.11. Reproducibility of SDMs Using Physiological Data from Omicron

We have seen that clinical data pertaining to Omicron reproduce the prediction of the SDMs. Reproducibility goes, however, beyond these clinical data. Experimental data that involve viral titration using bronchial and lung tissues have been published by a team at Hong Kong University [42,43]. In our previous publication [34], we showed that snippets of data coming out of HKU correlated considerably with the SDM predictions but, unfortunately, at the time of the writing of that previous paper, full data were not yet available, which resulted in statistical insignificance due to the small sample size [37,42]. In this paper, we will show that not only do the full HKU data [43] correlate with SDM predictions with statistical significance but also help us gain insights into some of SDM predictions.

**Figure 6:**
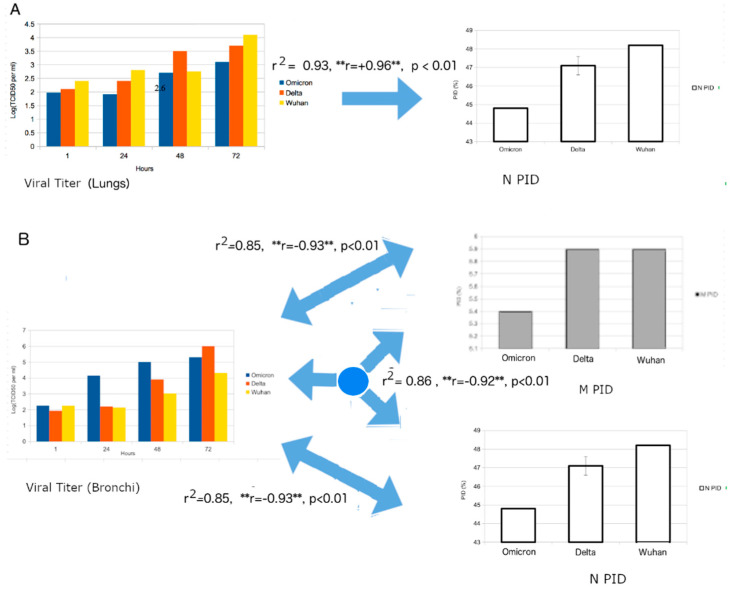
Regression Analysis of Viral Titer and corresponding PID_N_ and PID_M_ values. (**A**) Strong positive correlation can be seen between the viral titer from human lung tissues and PID. Regression model: VT = A × PID_N_) + B × Time + B, where VT = Viral Titer, A,B = Coefficients, C = Y-Intercept. (**B**). A strong negative correlation is seen between viral titer from human bronchial tissues and PID_N_ and PID_M_ values. (Regression model: VT = A × PID_M_ + B × PID_N_ + C × Time + D where VT = Viral Titer, A,B,C = Coefficients, D = Y-Intercept.) The figure is a statistical extension of the data from [43] using disorder information. “**” denotes the importance of a negative or positive correlation (r). The significance of this will be revisited in the next subsection.

The HKU group obtained bronchial and lung tissues separately and infected each with a variety of variants including Omicron. The viral titers were carefully measured at each stage. It was found that the viral titer of Omicron was higher in the bronchial tissues in comparison to that in the lung tissue (Figure 6). This was especially so when compared to the Wuhan-Hu-1 strain and other variants, including Delta. The authors attribute the results to the differences in ACE-2 on the different cell types [43]. The S protein of the virus binds to ACE-2 during viral entry. It is assumed that the differences provide for better entry. Unfortunately, they did not look into other possible explanations that involve other proteins.

### 3.12. Biochemical and Physiological Differences in Lungs and Bronchial Tissues: Mucus and Anti-Microbial Enzymes

We argue, however, that in order to fully appreciate the significance of their results, a more rigorous understanding of the complex physiology involved is necessary. The respiratory system is made up of two zones: the conducting and respiratory zones [94,95,96,97]. The conducting zone includes mucus-secreting cells and a network of mucociliary escalators that serve to move harmful foreign particles such as viruses away from the lungs. In yet another mode of action, as already mentioned, mucus contains anti-microbial enzymes that can damage the proteins, glycoproteins or RNA/DNA of pathogens and other foreign materials. The respiratory zone, on the other hand, includes alveoli, where gaseous exchange takes place. It is for these reasons that the upper and lower respiratory systems are different at the biochemical and cellular levels [40,96,97]. The bronchus is made up of three types of main cells. They are cilia cells, goblet cells, and basal cells. Cilia cells are covered with mucus, whereas goblet cells secrete mucus [97]. The lungs, on the other hand, are mainly made up of alveolar type I cells (AT1), macrophages, and type 2 pneumocytes (AT2) [96]. Unlike bronchi, much of the lung and bronchioles (part of the lungs) are devoid of mucus-secreting cells, and AT1 cells secrete surfactant instead [96,97]. In fact, experimental studies have shown that tissues from the upper respiratory regions have 10 times the amount of lysozyme, an anti-microbial enzyme, found in samples from the lower respiratory region [40].

The knowledge from both the physiology of the human respiratory system and the SDMs provides for a more adequate understanding of the significance of the findings by Hui et al. [43]. As noted above, physiology suggests that the amount of mucus and anti-microbial enzymes in the lung tissues is lower than that of the bronchi. SDM analysis, on the other hand, suggests that the Omicron has lower PID_N_ and PID_M_ values than all variants including the Wuhan strain and Delta. Therefore, according to the SDMs, Omicron is able to resist the anti-microbial enzymes but will produce fewer viral particles in the lungs. Given that there are more anti-microbial enzymes in the bronchial system, SDMs predict that there will be fewer viral particles in the lung tissue than in the bronchial tissue. This has been corroborated with statistical significance by the HKU group by showing that the viral titers in the lungs are lower than the ones for the bronchial samples when compared to the other variants [43].

### 3.13. Strong Positive and Negative Correlations in Lung and Bronchial Tissues, Respectively: Absence and Presence of Mucus

In order to obtain even more convincing results, we attempted to correlate viral titers of viruses to their PIDs (see Figure 6). Figure 6A shows a strong correlation (r^2^ = 0.9, *p* < 0.01) between the viral titer of the lung tissue with the PID_N_, while Figure 6B illustrates the strong correlation (r^2^ = 0.9, *p* < 0.01) between the viral titer and the PID_N_ and PID_M_ values, with both PID_N_ and PID_M_ as independent variables. Something odd was noticed when we carefully analyzed the results. The viral titer of the lung sample had a positive correlation with PID_N_, but the viral titer of the bronchial tissues had negative correlations with both PID_N_ and PID_M_. While it is odd that negative correlations were seen for both PID_N_ and PID_M_, the overall correlations further corroborate the SDM predictions. A positive correlation between the viral titer of lung tissue and PID_N_ means the greater the disorder of N, the greater the amounts of viral particles recovered in the lung tissues, and, therefore, Omicron should have the fewest viral particles recovered since it has the lowest PID_N_ among the variants. A negative correlation between the viral titer of bronchial tissues and the PID_M_ values confirms the SDM prediction that the viruses with harder M (low PID_M_) are able to resist the greater pressure of the anti-microbial enzymes found in the bronchus.

The puzzling thing, however, is the existence of a negative correlation between the viral titer of bronchial tissues and PID_N_. After a search of our data on other viruses from past research, this result should not be surprising at all but, rather, it should add to our knowledge of SARS-CoV-2. In the past, we have noticed a large variety of viruses in contact with saliva that have both hard inner and outer shells, which implies that the inner shells do also protect the virion from damage. Examples include many flaviviruses, rabies virus and EIAV. Looking into the data from Hui et al. [43] and our PID_N_ values, it can be seen that Delta’s PID_N_ is 47.1 + 0.8, which is slightly lower than the corresponding values found in other SARS-CoV-2 variants but not Omicron. Delta could have strengthened the negative correlation in the case of PID_N_ and bronchial tissues.

### 3.14. Confirmation of SDM Predictions for Pangolin-CoV: Attenuation in 2017 Pangolin-CoV

**Figure 7:**
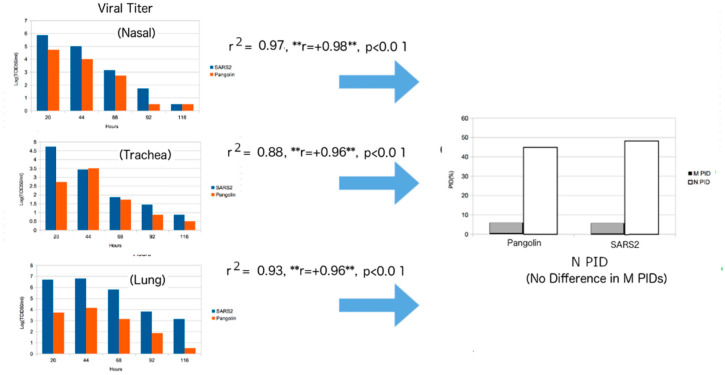
Regression Analysis of Viral Titer of pangolin-CoV/SARS-CoV-2 and PID_N_ and PID_M_ Values. Phylogenetic tree using M protein. The figure is a statistical extension of the data from [44] using disorder information. The regression model used is: VT = A × PID_N_ + B × Time + C, where VT = Viral Titer, A,B = Coefficients, C = Y-Intercept. ** Notice that the correlation(r) has not changed with different samples, unlike the HKU experiment in Figure 6.

Before the known arrival of Omicron, the SDMs predicted that the 2017 pangolin-CoV is attenuated with slower spread potential [35]. When Omicron came into the light, it was found that the 2017 pangolin-CoV had a PID_N_ almost identical to that of Omicron, even though Omicron has a much lower PID_M_. Once again, the SDM predictions were confirmed. The reason that the 2017 pangolin-CoV was first identified as attenuated is that it PID_M_ is lower than that of all SARS-CoV-2 variants with the exception of Omicron. It was also predicted to have a somewhat slower spread because, while its PID_N_ is lower, its PID_M_ is about the same as that of SARS-CoV-2, again with the exception of Omicron. Omicron is seen as more infectious as the result of its unusually low PID_M_. At least two laboratories [44,45] have also experimentally discovered that the 2017 pangolin-CoV is attenuated, as seen in Figure 7.

### 3.15. Strong Positive Correlations for Pangolin-CoV with PID_N_ in Hamster: Mucociliary Escalator in the Animal Model

An attempt to correlate the viral titer to PID_N_ was successful, with high correlations for the samples obtained from the nasal region, trachea and lungs. A seeming contradiction with the HKU data can, however, be found when we observe strong positive correlations in the nasal and tracheal samples (Figure 5), in contrast to the negative correlation found in HKU’s bronchial sample (Figure 6). For this, we need to understand that the experiments of Hui et al. [43] and Guo et al. [44] were conducted differently. Hui et al. were able to obtain bronchial and lung tissues separately and infect each with SARS-CoV-2 [43], whereas Guo et al. infected live hamsters with SARS-CoV-2 and pangolin-CoV [44]. Hui et al. measured viral titers by removing parts of their samples at each stage [43], while Guo et al. euthanized the hamsters to remove the various samples at various stages [44]. These differences had important effects on the titration data, because the physiology indicates the importance of a mucociliary network that is used to move foreign matter away from the lungs. Therefore, the full effects of the escalator are likely to have taken place in the case of the experiment of Guo et al., not Hui et al. Therefore, the positive correlations in the nasal and tracheal samples arise from the higher production of viral particles, especially in the lungs due to higher PID_N_, that are subsequently transported to the nasal and tracheal regions by the mucociliary escalator (Figure 7). Again, SDM predictions are experimentally supported, even to the smallest detail.

## 4. Discussion

### 4.1. Lingering Mysteries and Incoming Data

From the beginning of the COVID-19 pandemic, there were many questions pertaining to the nature of the virus. Many of these remain unaddressed or partially addressed. Where did the virus come from? What is its intermediary host? Do pangolins or bats serve as its intermediaries? Why is the virus so contagious? Where did Omicron come from? Since there about 50 mutations in Omicron [18], how did Omicron acquire such a large number of mutations in such a short time period? Why is Omicron mild? Is it really inherently mild? Why is Omicron more contagious than other SARS-CoV-2 variants? Omicron remains shrouded in mystery, as its mutations are different from the mutations seen in other known variants. A question, then, is: Where was Omicron hiding all along? These are some of the many Omicron mysteries, and SDMs have specific answers or hints to many of these questions. Many of these questions have been addressed in previous publications, but we believe that many of the questions can be addressed with greater confidence given the newly retrieved data, which include more complete details on Omicron viral titration using lung and bronchial tissues, experimental results on animal models pertaining to the pangolin-CoV attenuation, and new information pertaining to the COVID-19-related bat-CoVs from Laos.

### 4.2. Contagiousness of SARS-CoV-2: The Hard Outer Shell (M)

Why is SARS-CoV-2 so highly contagious that it became a pandemic? A popular answer is the affinity of the SARS-CoV-2 S protein to the human ACE-2, which is essential to viral entry [98,99,100,101,102,103,104]. Clinical studies show, however, that COVID-19 patients shed large amounts of viral particles [92]. This could mean that even if the affinity studies are valid, they may not be relevant. A more plausible or relevant explanation that is consistent with the clinical data, lies within the SDMs. There are two important factors pertaining to the SDMs as applied to COVID-19. One is the disorder status of the outer shell (PID_M_), and the other is that of the inner shell (PID_N_). A more rigid M (lower PID_M_) is associated with more protection for the virus, especially against the anti-microbial enzymes found in saliva and mucus. On the other hand, higher levels of the N disorder (higher PID_N_) are associated with higher virulence, since N is intimately involved in the replication process [88].

### 4.3. Uniqueness of COVID-19 Related Viruses, Abnormally Low PID_M_ and the Pandemic

Upon the COVID-19 outbreak, we immediately noticed something very odd about SARS-CoV-2. Its PID_M_ was among the lowest in the entire CoV family, as seen in Table 2 and Figure 1. As we carefully looked over our CoV database, we found that this abnormally hard outer shell is associated with burrowing animals such as rabbits [22,36], and there are very few such viruses. Using our knowledge from the CoV-transmission SDM, we came to the realization that the hardness had been acquired via exposure to buried feces. It was later shown that that pangolin-CoVs are closely related to SARS-CoV-2 [22,35,36,37]. Pangolins are also burrowing animals. As more data came, it became apparent that low PID_M_ values are the hallmark of all COVID-19-related CoVs (Figure 2B and Table 3). While it requires a specific evolution involving a burrowing animal for the virus to attain such a low PID_M_, apparently this low PID_M_ provides greater fitness in the spread of the virus among humans, as the PID_M_ currently shows no sign of increase among the variants.

When the CoV transmission-shell disorder model was first designed, we could find very few, if any, CoVs with a low PID_M_ comparable to that of SARS-CoV-2 (see Table 2). It is not a coincident that with the exception of the COVID-19-related viruses, there are very few CoVs with such a hard outer shell, and that there has been no CoV pandemic on the scale of COVID-19 in the past. In order for a CoV to attain such low PID_M_, it needs to undergo a specific evolution that potentially involves a burrowing animal and buried feces. This hard outer shell is essential for the kind of contagiousness necessarily for spread in a human pandemic, since its resistance to the nasal and oral anti-microbial enzymes allows the virus to shed in large quantities. Ironically, farm animal pandemics involving CoVs are actually common, but non-human animal-CoVs rely mainly on the fecal–oral transmission for their spread, while human pandemics, such as COVID-19, on the other hand, rely on respiratory transmission for their faster spread. This is the case in TGEV, where the virus typically moves very rapidly in pigs that are bred in close proximity to each other [26,29].

### 4.4. Pangolin-CoVs Have More Diverse Characteristics That Resemble SARS-CoV-2

While bat-CoV genes have been found to have the greatest homology to SARS-CoV-2, genetic similarities do not tell the whole story, as recombinations often occur between viruses. Furthermore, our data indicate that pangolin-CoVs share a more special relationship with SARS-CoV-2 for three reasons. Firstly, the potential connection of the SARS-CoV-2 to burrowing animals tells us that there is a likelihood of an intimate relationship. Secondly, the PID_N_ values of pangolin-CoVs offer a more diverse range of values that matches that of SARS-CoV-2. With the availability of the genetic sequences of the Laotian bat-CoV and RaTG13, we now have sufficient samples to compare the PID_N_ values. We see, for example, that the Omicron PID_N_ is 44.8%, which is similar to that of the 2017 pangolin-CoV, while none of the bat-CoV PID_N_ values came close. How did pangolin-CoVs achieve such low PID_N_? A plausible answer is that pangolins may have been harboring a reservoir of COVID-19 viruses for years and, maybe, decades, perhaps, more so than bats. It is also likely that during the decades or years, COVID-19-related viruses have been moving in and out of the species through interspecies transmission. Thirdly, we shall see that pangolins, through their behavior, offer a venue for the attenuation of the virus as seen in Omicron.

The virulence-inner shell disorder suggests that there is a correlation between the inner shell disorder (PID_N_) and the level of virulence for a virus. We are, therefore, able to explain why SARS-CoV-2 is more virulent than SARS-CoV-1: SARS-CoV-1 N (PID_N_: 50%) is more disordered than SARS-CoV-2 N (PID_N_: 48%). Before the discovery of Omicron, a comparative analysis allowed us to project that the 2017 pangolin-CoV stood out as an attenuated virus with a lower virulence. The discovery of Omicron actually supported these predictions, because Omicron is clinically shown to be milder than the other variants, and its PID_N_ is very similar to that of the 2017 pangolin-CoV. Omicron is, however, more contagious than other known variants. This also supports the SDM prediction that the 2017 pangolin-CoV would likely have slower potential spread if it had entered humans. This prediction was based on the fact that it has a lower PID_N_ than the non-Omicron SARS-CoV-2 and yet has a similar PID_M_. This is in sharp contrast to similar PID_N_ values for Omicron and 2017 pangolin-CoV, but a lower Omicron PID_M_ than 2017 pangolin-CoV and other variants.

### 4.5. Statistical Study Adds to Our Knowledge of Inner Workings of SDMs and Respiratory Physiology

Pangolin-CoV and Omicron studies have not only supported our predictions but strengthened our arguments by pointing to the precise mechanisms in the roles of N and M in attenuation and human contagiousness. Our statistical extension of these studies to the SDMs provides greater insights into the application of SDMs in the study of COVID-19. The statistical extension forces us to apply knowledge of the physiology of the respiratory system to SDMs. Physiology tells us that the lung cells are mostly devoid of mucus, unlike the bronchi in which mucus-producing cells play important roles [94,95,97]. The SDMs, on the other hand, stipulate that the harder shells, especially a harder outer shell (lower PID_M_), will make the virus more resistant to the anti-microbial enzymes found in the mucus, and, in the event of an absence or lack of anti-microbial enzymes, the quantity of virus copies will be dependent on the N disorder (PID_N_). Using both physiology and SDMs, it is, therefore, predicted that more viral copies will be found in the viral titration using bronchial tissues than the one using lung tissues. The confirmation of the results is reflected in the high positive correlations found between PID_N_ and viral titer for the lung tissues. Similarly, reproducibility can be found when both PID_N_ and PID_M_ were observed to be negatively correlated with the viral titer. The negative correlation for PID_N_ reflects the probability that a harder inner shell (N) also plays some role in protecting the virion, namely RNA. Such a protective role has been observed in other viruses, such as rabies virus, some flaviviruses and equine infectious anemia virus (EIAV) [24,25,26,29,31,33].

Similar confirmation of the SDMs on a slightly different aspect can be found in the experimental data that show the attenuation of the 2017 pangolin-CoV. This time, it involved Syrian hamsters as an animal model. Keeping in mind that the PID_M_ of pangolin-CoV-2017 is similar to that of non-Omicron SARS-CoV-2, and also that the PID_N_ values of pangolin-CoV and Omicron are virtually identical, there are positive correlations between the PID_N_ values and viral titers from the lungs and upper respiratory system. The positive correlations are consistent with SDMs and the mucociliary escalator network.

### 4.6. Laotian Bat-CoVs and Omicron Are Closely Related to Pangolin-CoVs

As we see, pangolin-CoVs have a much closer relationship to SARS-CoV-2 than CoVs from bats. There is a closer range of resemblance of PID_N_ values between pangolin-CoVs and SARS-CoV-2. As seen in Table 2 and Table 3 and Figure 4, Omicron is largely responsible for this enigmatic pattern and trend. Part of the enigma has to do with the large number of mutations acquired by Omicron, not seen in other variants. Our phylogenetic analysis using M tells us that Omicron did not descend from the Wuhan-Hu-1, but rather from one of its ancestors. Figure 3C also shows that Omicron is likely to be more closely related to the pangolin-CoVs. Not only that, but RaTG13 and the Laotian bat-CoVs have close relationships with pangolin-CoVs. There are speculations that Omicron had been hiding in immune-suppressed HIV patients [23]. According to SDMs, however, it is likelier that Omicron had been hiding in a burrowing animal, such as pangolins. The oral–fecal potentials of pangolins provide a more optimal environment for the hardening of not just the outer shell but also the inner shell, as both enhance the chances of the virus surviving in feces buried for a long time. In the process, the result is an attenuated virus that spreads more rapidly when it re-enters the human population.

### 4.7. SDMs Hint at the Differences in the Evolution of Various Burrowing Animals

Before COVID-19, we were only able to find two CoVs that had exceptionally low PID_M_ values. They are rabbit-CoV and HCoV-HKU1, both of which are associated with burrowing animals. While we understood the role of PID_N_ in the categorization of CoVs in the CoV transmission-shell disorder model, we were not quite able to figure out the role played by M due to the low number of low PID_M_ samples, even though our statistical models did indicate the significance of PID_M_. It was only with the arrival of COVID-19 and, especially, Omicron that we were able to fully grasp the relevance and inner workings of M. Rabbit-CoV has a PID_M_ and PID_N_ of 5.3% and 53%, respectively. The odd thing is that the rabbit-CoV PID_N_ is higher than that of SARS-CoV-2. This discrepancy can be explained by the differences in the behaviors of rabbits and pangolins. Pangolins flick their sticky tongues to trap and eat ants on the ground that could be contaminated with feces, while rabbits eat leaves that are usually above the ground even though they live in burrows. As a result, higher PID_N_ and more fecal–respiratory transmission potentials may be required for rabbit-CoV.

### 4.8. The Mysterious HCoV-HKU1 and Mice: Dual Evolution of Mice

HCoV-HKU1 has occasionally caused outbreaks among humans that are sometimes associated with hepatitis, but its outbreaks are nothing like that of the COVID-19 pandemic [85,86]. Its shell PIDs are just as mysterious as the virus. It has one of the lowest PID_M_ values (4.8%), but it has also the lowest PID_N_ (~37%) seen in any CoV. The abnormally low PID_N_ could explain why it never caused a pandemic like COVID-19. Even though it has been in humans since 2003, we still do not know where this mysterious virus came from. A hint can be found in phylogenetic studies that show that it is closely related to mouse and rat CoVs. Curiously though, murine hepatitis virus has a moderate PID_M_ (8%), unlike HCoV-HKU1 or even SARS-CoV-2. This can be explained by the complex evolution of mice and rats. Rats and mice have dual evolutions of living in burrows and in human homes. They have evolved with humans for centuries to live with humans in villages and towns, even though some species live in burrows in rural or forested settings [87]. Some scientists have suggested that Omicron was hiding in mice before showing up among humans [105]. Given the dual evolution, this is possible, but we are skeptical, as mice may not provide the optimal environment for attenuation, unlike pangolins.

### 4.9. Pangolins Provide a More Optimal Evolutionary Environment for Attenuation and More Efficient Human Spread

We argue that the pangolin offers a more optimal environment for CoV attenuation and more efficient spread than do mice, rats, rabbits or immuno-compromised HIV patients, because of its unique fecal–oral behaviors as described above. The statistical study performed in this paper infers that the virion is not only protected by a more rigid M but also by a more rigid N (lower PID_N_). This also implies viruses with harder N and M are likelier to remain active longer in buried feces. A harder N is, however, associated with lower virulence under the virulence-inner shell model. This feature and characteristic are exactly what have been found in Omicron. The fact that the phylogenetic tree using M (Figure 3B,C) shows that Omicron is not a descendant of Wuhan-Hu-1 but is more closely related to pangolin-CoV adds to the evidence. This may explain the reason why Omicron is able to accumulate so many mutations, a question that has puzzled scientists who assumed otherwise, given the proof-reading mechanism of CoVs.

### 4.10. SDMs Do Not Contradict the Zoonotic Transmission Events at the Wuhan Market

Much effort has been made to show that COVID-19 has a natural origin. This includes at least two prominent papers [1,2] that show that the Wuhan outbreak arose from a zoonotic transmission from the animals in the market. While our paper does provide evidence that could support a natural origin of COVID-19, the data presented in our paper could be viewed as a contradiction to the conclusion that the outbreak in Wuhan had an immediate animal origin. Such would be the wrong interpretation of our paper. While our data could suggest that COVID-19-related viruses could have moved back and forth among humans and various animals, there is nothing in our data to suggest the presence of a COVID-19 in humans immediately prior to the Wuhan outbreak. It is possible that a COVID-19 virus did enter the human population a few years before the Wuhan outbreak, before becoming temporarily extinct, but not before a reverse transmission back to its reservoir. The idea that COVID-19-related viruses have been moving in and out of several species with pangolins as the main reservoir should be considered very plausible, since SARS-CoV-2 is highly adapted to multiple species [101,104]. Applying the theory of evolution, it is most likely that the virus gained adaptability to multiple species by numerous incursions into many species over a long period of time while retaining a main reservoir in which it would gain more adaptability with each incursion via reverse transmission. Why should humans be exempted from such incursions in the past, especially given that the virus is highly adapted to humans [101,104]?

### 4.11. Other Reproducible SDM Predictions Not Mentioned as Applied to SARS-CoV-2

We also need to understand that the results presented in this paper are not the only evidence of the reproducibility of SDMs by other independent laboratories throughout the world. Some of them have already been mentioned in our previous publications. For example, Ogando et al. [93] has found that SARS-CoV-1 produces more viral particles than SARS-CoV-2 when VERO-E6 cells are infected. Given that it has been clinically shown that COVID-19 patients shed large amounts of the virus, it can only mean that SARS-CoV-2 is producing fewer viral particles in vital organs but sheds more nasally and orally, as M is more resistant to the anti-microbial enzymes found in saliva and mucus. Another group, Riddell et al. [106], reported that SARS-CoV-2 lasts many times longer on surfaces away from sunlight than do many CoVs such as TGEV (PID_M_: 14%) and MHV (PID_M_: 8%), which have a higher PID_M_ than SARS-CoV-2 (PID_M_: 5.8%). Furthermore, Omicron was recently seen as being more resilient on the surface than other variants, as predicted by SDMs [107].

## 5. Summary and Conclusions

While the predictions of the SDMs have been shown to be highly reproducible in even finer detail by experimental and clinical studies, this paper reports and analyzes a series of data that were previously incomplete or absent at the time of our previous publications. We extended the HKU experiments pertaining to the Omicron infection of lung and bronchial tissues to show not only that the experiment was statistically consistent with the SDMs but also showed us an aspect of the SDMs that we did not know was applicable to COVID-19. The statistical differences can be attributed to hardness of the shells of the various SARS-CoV-2 variants that protect their virions from damage arising from the anti-microbial enzymes in mucus, which is present in the bronchial tissues but largely absent in the lung tissues. The biochemical difference can be traced to physiology. While the HKU experiment was related to infected tissues in vivo, the pangolin-CoV experiment was based on an animal model using Syrian hamsters. Our statistical extension of the latter experiment bore results that reflect this difference.

In the the case of the HKU experiment, a positive correlation was seen between N PID and viral titers of lung tissues, while a negative correlation was seen between N-M PIDs and viral titers of bronchial tissues. However, in the case of the pangolin-CoV experiment, there were positive correlations between N PIDs and viral titers of all samples from lung, trachea and nasal samples (the M PID of pangolin-CoV-2017 is virtually the same as the M PIDs of all SARS-CoV-2 variants except Omicron). This basically confirms the SDM prediction that pangolin-CoV-2017 is attenuated (attenuation seen in the experiment) by its lower N PID. It is also amazing that the statistical result also detected the action of the mucociliary escalator, which basically tells us that the mucus and the fluid in the entire respiratory system move the virus particles away from the lungs and towards the upper respiratory system. This accounts for the differences in correlations for the two separate experiments. It should also be reiterated that the pangolin-CoV experiment validated the virulence-inner shell disorder model (one of the three SDMs), which stipulates that the more disorder in the inner shell protein (N, in the case of COVID-19), the more virulent the virus is, because the greater disorder increases the efficiency of the protein–protein/RNA/DNA binding necessary for quicker viral replication.

The third set of results, not previously seen, involves the shell disorder analysis of COVID-19 related bat-CoVs that were recently discovered in Laos. The addition of the Laotian bat-CoV disorder data increased our sample size of COVID-19 bat-CoV such that we were able to observe that even with the small sample size of pangolin-CoVs, the N PIDs of pangolin-CoVs are more diverse and match more of those of SARS-CoV-2 variants, especially Omicron. Furthermore, a phylogenetic study using the highly conserved M (among COVID-19 CoVs) showed a close relationship of pangolin-CoVs to bat-CoVs, which implies the presence of recurring interspecies transmissions over a long period. This could suggest that the way the ancestors of SARS-CoV-2 acquired their multi-species adaptation evolutionarily was by having regular incursions into other species, as exemplified by the phylogenetic cross-species transmissions we observed.

Greater disorder of the N protein induces greater and faster replication of the virus, especially in vital organs, whereas a harder outer shell (lower disorder levels of M or lower PID_M_) provides greater protection against the anti-microbial enzymes found in mucus and saliva. These basic tenets enable SDMs to make some bold but highly reproducible predictions. The models are thus able to predict that SARS-CoV-2 is less virulent but more contagious than SARS-CoV-1 because of the differences in their PID_M_ and PID_N_. These are consistent with what is clinically known about the 2003 SARS and COVID-19. Before the known arrival of Omicron, the 2017 pangolin-CoV was identified as attenuated based on its low PID_M_, and it was proposed that it was likely to be slower-spreading if it entered the human population. The attenuation and slower spread makes it ideal for a cryptic spread, as it could be easily mistaken for a mild cold by the medical community. Upon arrival in the human population, Omicron was found to be clinically milder but more infectious. A glance at its PID_M_ and PID_N_ tells us that the PID_N_ is almost identical to that of the 2017 pangolin-CoV, while its PID_M_ is much lower than that of the 2017 pangolin-CoV, which is similar to other variants. While clinical observations of Omicron validate the SDM predictions pertaining to both Omicron and the 2017 pangolin-CoV, the Omicron and pangolin-CoV experiments of Hui et al. and Guo et al. supported the COVID-19 mechanisms of attenuation and spread as laid out by the SDMs.

The low PID_M_ that can be found in all current COVID-19-related CoVs is associated with burrowing animals, namely pangolins. Pangolins, through their fecal–oral feeding behaviors via buried feces, provide a more optimal environment for the hardening of both inner (N) and outer (M) shells. Our phylogenetic study using M also included data from COVID-19-related bat-CoV from Laos. As already mentioned, the phylogenetic tree revealed that the COVID-19-related bat viruses are clustered together with pangolin-CoVs. Omicron is also interestingly clustered more closely with pangolin-CoVs than with the other SARS-CoV-2 variants. Unlike the bat-CoV, the pangolin-CoV PID_N_ range is more diverse and more closely resembles those found in SARS-CoV-2 variants including Omicron, which allows us to speculate that pangolins, not bats, served as the main reservoir. The phylogenetic tree also suggested that the interspecies transmissions had taken place regularly over many years or decades before the beginning of the COVID-19 pandemic, which supports the possibility of slow cryptic incursions into humans from pangolins in 2017 or before. It is also possible that these intruding ancestral viruses withdrew back into their main reservoirs, only to return later with greater human adaptation.

While the prevailing current explanation for the contagiousness of SARS-CoV-2 lies in the S protein, clinical evidence seems to indicate the large amounts of shedding by COVID-19 patients [92,108,109] are more consistent with the paradigm provided by SDMs. A reason for this inconsistency may have to do with the limitation of the attempts to link any single protein such as the S to every characteristic of the virus. While S is the most studied CoV protein since it is involved in many crucial roles such as viral entry and antibody recognition of the virus, it can be argued that it is not necessarily the most important protein and certainly not the only CoV protein. It is, therefore, vital that we also look at other CoV proteins such as the M and N that are not as well-studied and play different roles for a more complete understanding of the behavior of the virus, and we are using SDMs via AI molecular tools to do that.

It is also important to note that the M and N disorders likely do not affect only the spread and attenuation of the virus, but also modify many other clinical aspects of COVID-19. One of these is the problem of long COVID-19, where the patient still suffers symptoms for months and years after recovery. It is possible that the hard M is preventing the immune system from eliminating the particles or proteins. This possibility, however, has not been looked at, despite its obvious importance.

## Figures and Tables

**Table 1:** The Shell Disorder Models (SDMs). There are three closely related models based on the principles of protein intrinsic disorder. Levels of disorder are measured at each shell of the virus.

Year of First Publication	Shell Disorder Model	Details
2008	Parent Viral Shape-shifter Model	A database of viral shell proteins was built. HIV-1, HCV and HSV were found to have abnormally high disorder at their outer shells. No effective vaccine is currently available for the three viruses.
2012	CoV Transmission SDM	Correlations were found between disorder levels in M and N proteins and the modes of transmission; i.e., fecal–oral and respiratory transmissions.
2015	Virulence-inner Shell Disorder Model	Positive correlations have been found for virulence of many viruses and their inner shell disorder. Viruses include DENV, EBOV, NiV and SARS-CoV-1/2.

**Table 2:** Prediction of the Levels of Fecal–Oral and Respiratory Transmission Potentials using PID_M_ and PID_N_ Values as Grouped by Categories. Data pertaining to COVID-19-related bat-CoVs from Laos have been added. (Taken in part from Biomolecules. 2022;12(5). © 2022 Goh et al, Published by MDPI AG, Open access under CC BY-NC-ND license. This is an Open Access article which permits unrestricted non-commercial use, provided the original work is properly cited. [21]).

Coronavirus	PID_M_	UniProt(U) Genbank(G) Accession Code (M Proteins) ^a^	PID_N_	UniProt(U) Genbank(G) Accession Code (N) ^a^	Group/Remarks
HCoV-229E	23	P15422	56	P15130	Group A
IBV(Avian) ^c^	10	P69606	56	Q8JMI6	Higher levels of respiratory transmission lower levels of fecal-oral transmission
Bovine	7.8	P69704	53.1	Q8V432(U)	Group B
PEDV (Porcine) ^c^	8	P59771(U)	51.7	Q07499(U)	Intermediate levels of respiratory and fecal-oral transmission
Canine (Resp.)	7	A3E2F6(U)	50.5	A3E2F7(U)	
HCoV-OC43	7	Q4VID2(U)	51	P33469(U)	
SARS-CoV-1	8.6	P59596(U)	50.2	P59595(U)	
HCoV-NL63	11	Q6Q1R9(U)	49	Q6Q1R8(U)	
Bats ^b^	11.2 + 5.3	A3EXD6(U)	47.7 + 0.9	Q3LZX4(U)	
MHV(Murine) ^c^	8	Q9JEB4(U)	46.8	P03416(U)	Group C
MERS-CoV	9.1	K0BU37(U)	44.3	K0BVN3(U)	Lower levels of respiratory transmission higher levels of fecal-oral transmission
TGEV(Porcine) ^c^	14	P09175(U)	42.41	P04134(U)	
Canine (Ent.)	8	B8RIR2(U)	40	Q04700(U)	
HCoV-HKU1 ^d^	4.5	Q14EA7(U)	37.4	Q0ZME3(U)	
SARS-CoV-2					Group D
[Wuhan]	5.9	YP009724393(G)	48.2	YP009724397(G)	High Respiratory and Fecal-oral Transmission Potentials
[Delta]	5.9	QUX81285(G)	47.1 + 0.5	QYM89845(G)	
[Omicron]	5.4	UFO59282(G)	44.8	UFO692871(G)	
Pangolin-CoV ^e^	5.6 + 0.9	QIA428617(G)	46.6 + 1.6	QIA48630(G)	
Rabbit-CoV	5.7	H9AA37(U)	52.2 6 ^e^	H9AA59(U)	
RaTG13	4.1	QHT63303(G)	48.5	QHR63308(G)	
Laotian Bat-CoV	6.0 + 0.2		48.3 + 0.2		
[Banal-52	6.3	UAY13220.1	48.2	UAY13225.1	
[Banal-103]	5.9	UAY13232.1	48.5	UAY13257.1	
[Banal0236]	4.1	UAY13256.1	48.5	UAY1326.1	

^a^ UniProt (U): [https://www.uniProt.org, access on 23 August 2022]; GenBank-NCBI (G): [https://www.ncbi.nlm.nih.gov/protein, access on 23 August 2022]. ^b^ Summary figures on bats. Further details on the bat samples can be found in Table 2. Four out of five bat-CoVs are in group B. High standard deviations are seen for PID_N_ and PID_M_ values as denoted by “±”. ^c^ MHV (murine hepatitis virus), IBV (infectious bronchitis virus), PEDV (porcine epidemic diarrhea virus, TGEV (transmissible gastroenteritis virus). ^d^ HCoV-HKU1 has one of the lowest PID_M_, which could qualify it for group D, but its PID_N_ is also abnormally low. Much is still not understood about HCoV-HKU1 [50,51]. For these reasons, HCoV-HKU1 is left in group C. ^e^ Details on the known existing pangolin-CoVs can be found in Table 3. Standard deviation is denoted by “±”.

**Table 3:** Details of the pangolin CoVs and bat CoVs PID_M_ and PID_N_ values and their sequence similarities with SARS-Co-V and SARS-CoV-2 as references. Using PID_N_, wo sub-variants of Delta were detected using SDM (Delta1, Delta2) [21]. Data from COVID-19-related bat-CoVs have been added. (Taken in part from Biomolecules. 2022;12(5). © 2022 Goh et al, Published by MDPI AG, Open access under CC BY-NC-ND license. This is an Open Access article which permits unrestricted non-commercial use, provided the original work is properly cited. [21]).

Coronavirus	Sequence Similarity M (%)	PID_M_ (%)	Accession: UniProt (U) GenBank (G)	Sequence Similarity N (%)	PID_N_ (%)	Accession UniProt (U) GenBank (G)
SARS-CoV-1	90.5	8.6	P59596(U)	90.5	50.2	P59595(U)
Civet-SARS-CoV	90.1	8.6	Q3ZTE9(U)	90.01	49.1	Q3ZTE4(U)
Laotian Bat-CoV		6.0 ± 0.2			48.3 ± 0.2	
[Banal-52		6.3	UAY13220.1		48.2	UAY13225.1
[Banal-103]	98.7	5.9	UAY13232.1	99.3	48.5	UAY13257.1
[Banal-236]	98.7	4.1	UAY13256.1	99.1	48.5	UAY1326.1
	99.1			99.3		
Pangolin-CoV		5.6 ± 0.9 ^a^			46.6 ± 1.6 ^a^	
2019	98.2	6.3	QIG55948(G)	98	48.7	QIG55953(G)
2018	97.7	4.5	QIQ54051(G)	93.8	46.3	QIQ54056(G)
2017 ***	**98.2**	**5.9**	QIA48617(G)	**94**	**44.9**	**QIA48630(G)**
				93.32	46.5	QIA48656(G)
SARS-CoV-2						
[Wuhan]	100	5.9	YP009724393(G)	100	48.2	YP009724397(G)
[Delta1]	99.1	5.9	QUX81285(G)	99.3	46.8	
[Delta2]	99.1	5.9	QUX81285(G)	99.1	47.5	QYM89997(G)
[Omicron] **	**98.7**	**5.4**	UFO59282(G)	**98.6**	**44.8**	QYM89845(G)
						UFO692871(G)
Bat-CoV		11.2 ± 15 ^a^	Q9JEB4		47.7 ± 0.9 ^a^	
RATG13	99.6	4.1	QHR63303(G)	99.1	48.5	QHR63308(G)
Bat 512	35.5	15.3	Q0Q463(U)	29.4	46.5	Q0Q462(U)
HKU3	91	7.7	Q3LZX9(U)	89.6	48	Q3LZX4(U)
HKU4	42.7	16.4	A3EXA0(U)	51.1	48.5	A3EXA1(U)
HKU5	44.7	11.8	A3EXD6(U)	47.9	47.1	A3EXD7(U)

^a^ Standard deviation is denoted by “±”. ** Attenuated strains detected. *** One of the two 2017 pangolin-CoV isolates was detected to be attenuated.

## Data Availability

All data are readily available as shown in the Material and Methods (Section 2).
